# Nitrification in Activated Sludge Exposed to Static Magnetic Field

**DOI:** 10.1007/s11270-017-3316-6

**Published:** 2017-03-06

**Authors:** Marcin Zieliński, Agnieszka Cydzik-Kwiatkowska, Magdalena Zielińska, Marcin Dębowski, Paulina Rusanowska, Joanna Kopańska

**Affiliations:** 1 Department of Environment Engineering, University of Warmia and Mazury in Olsztyn, Warszawska 117, 10-720 Olsztyn, Poland; 2 Department of Environmental Biotechnology, University of Warmia and Mazury in Olsztyn, Słonczena 45G, 10-709 Olsztyn, Poland

**Keywords:** *amoA* gene copy number, Nitrification, Static magnetic field

## Abstract

The study investigated wastewater treatment in an aerobic reactor with activated sludge exposed to static magnetic field (SMF) with mean induction of 8.1 mT. The efficiency of chemical oxygen demand removal was about 90% in a control reactor and an SMF-exposed reactor. Although the nitrification efficiency was higher than 95% in both reactors, the activity of ammonia-oxidizing bacteria was higher in the SMF-exposed reactor. This resulted in shortening of nitrification time to 4 h compared to 8 h in the control reactor. Higher number of ammonia-oxidizing bacteria in the SMF-exposed reactor might result from increased oxygen penetration into the liquid exposed to SMF, which favored growth of these bacteria. The results indicate that SMF enhanced nitrification, the most sensitive process from the biological nitrogen transformations. SMF influenced the overall biomass content that was 14% higher in the SMF-exposed reactor than in the control reactor.

## Introduction

Biological nitrogen removal from wastewater by nitrification and denitrification is one of the most common treatment processes. Ammonium is oxidized to nitrites by ammonia-oxidizing bacteria (AOB), and nitrites are further oxidized to nitrates by nitrite-oxidizing bacteria (NOB). Nitrification limits the rate of nitrogen removal from wastewater and is one of the most sensitive biological processes during wastewater treatment. To ensure an efficient nitrification, the effective aeration is needed. Aeration costs represent the largest share of total operating cost of wastewater treatment plants, because oxygenation of wastewater should occur efficiently (Rosso et al. 2008).

In recent studies, it was proved that magnetic field exerts a positive effect on many properties of fluids, i.e., it changes the polarization and electric charge and sets the particles. In most of studies, magnetic field is used for separation of solids (mainly activated sludge) from effluent (Zieliński et al. 2014). It was also observed that magnetic field could affect the bacterial growth and metabolism. Okuno et al. (1993) proved that magnetic field is not lethal to microorganisms but might influence a growth rate of bacteria. This effect depends on the intensity and frequency of the field, the static or oscillating character of the field, the wave form, the type of exposed cells, and the condition of these cells (Dini and Abbro 2005; Yadollahpour et al. 2014). Jung et al. (1993) reported that magnetic field with a strength of 9 mT did not have any effect on phenol biodegradation, magnetic field with a strength of 17.8 mT had a positive effect, while magnetic field of about 54 mT lowered the removal efficiency and growth of microorganisms. Magnetic fields with inductions of 150 and 350 mT improved phenol biodegradation by immobilized activated sludge (Jung and Sofer 1997). Yavuz and Çelebi (2000) changed direct current magnetic field strength and observed that pulsed magnetic field did not change significantly the activity of the activated sludge while alternating current caused a slight decrease in this activity.

In preliminary studies, the authors have observed that magnetic field enhanced the efficiency of liquid aeration. The oxygen capacity (OC) of water treated by static magnetic field (SMF) of 27 mT was 93 g/(m^3^ h), and of water untreated, it was 61 g/(m^3^ h) (unpublished data). Krzemieniewski et al. (2003) have showed an increase in oxygen saturation of the liquid exposed to magnetic field and the elimination of gases with higher atomic masses. The authors have also noted that the introduction of magnetic field might reduce the occurrence of carbon dioxide and hydrogen sulfide in municipal sewage. In the liquid subjected to magnetization, an increase of oxygen content might result in proliferation of aerobic microorganisms and, hence, a greater degree of decomposition of organic substances (Krzemieniewski and Filipkowska 1998). Although there are some studies on the effect of magnetic field on wastewater treatment with activated sludge, there is no data that combine the efficiency of wastewater treatment with the number and activity of bacteria in activated sludge exposed to magnetic field.

In this study, the effects of magnetic field with induction of 8.1 mT on wastewater treatment and the activity and abundance of AOB and overall microbial community of activated sludge were assessed.

## Materials and Methods

### Experimental Setup

**Fig. 1:**
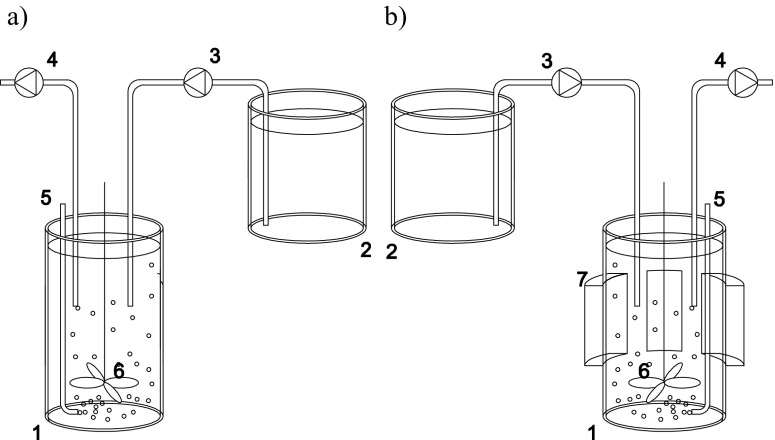
Scheme of a research station in the control reactor (**a**) and the SMF-exposed reactor (**b**): *1* SBR, *2* retention tank, *3* dosing pump, *4* drain pump, *5* aeration system, *6* mixing system, *7* magnetic liquid activators

**Fig. 2:**
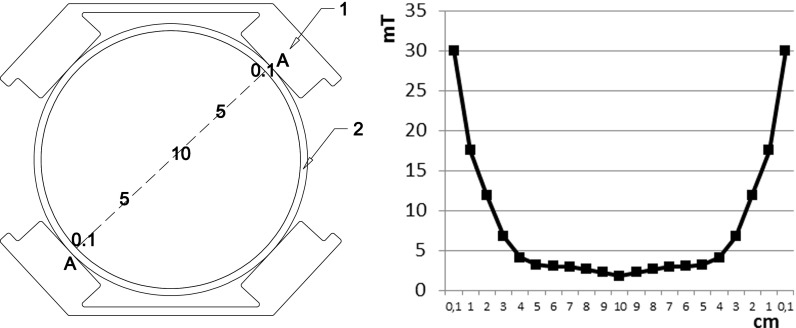
Distribution of magnetic field in the reactor along section *A*–*A*: *1* magnetic fluid actuator, *2* glass wall of the reactor

The study was done in the batch bioreactor BioFlo 310 (New Brunswick) with a working volume of 4 L operated at a temperature of 20 ± 2 °C. The reactor cycle lasted for 24 h and consisted of filling (15 min), aeration (23 h), settling (30 min), and decantation (15 min). The intensity of aeration was 200 L/h. The volumetric exchange ratio was 25%/cycle. The synthetic wastewater was prepared according to Weinberger’s method (PN-C-04616/10 1987) from peptone (0.6 g/L), starch (1.5 g/L), urea (0.9 g/L), anhydrous sodium acetate (0.3 g/L), calcium chloride (0.21 g/L), potassium chloride (0.21 g/L), and magnesium sulfate heptahydrate (3.075 g/L). The concentrations of pollutants in wastewater were 1000 ± 150 mg/L of chemical oxygen demand (COD), 113.5 ± 10 mg/L of total nitrogen (TN), 7.3 ± 2 mg/L of N-NH_4_^+^, and trace amounts of N-NO_3_^−^ and N-NO_2_^−^. Activated sludge from an aerobic chamber from wastewater treatment plant in Olsztyn (Poland) was the inoculum. The organic loading rate was 0.043 g COD/(g mixed liquor suspended solids (MLSS) day). The HRT was 4 days and the SRT was 590 days in the control reactor and 912 days in the SMF-exposed reactor. To determine the influence of SMF on organic and nitrogen compound conversions, the experiment was done in two series. In the first series, wastewater was treated in the control reactor. In the second series, in a lower part of the bioreactor, the magnetic fluid actuator (MFA), built of two parts forming a ring, was placed (Fig. 1). Permanently magnetized ceramic frits in MFA emitted SMF. The parameters of the MFA were width of the ring of 65 mm, height of the individual ceramic magnet of 45 mm, weight of the individual ring of 1.25 kg, and nominal diameter range of 90–110 mm. The induction inside the reactor was measured using the digital Gaussmeter LZ-641H (ENES Magnesy). The gaussmeter was immersed in the liquid, starting from the edge of the reactor to the opposite side. The distance between measurement points was 1 cm. The magnetic induction decreased with distance from the source of field from 30 mT directly at a source of field to 1.8 mT in the center of the reactor (Fig. 2). Due to the complete mixing of the reactor content during the treatment process, the average value of the magnetic induction of 8.1 mT was assumed.

The adaptation period lasted, about a month, until the values of pollutants in the effluent did not vary more than 10% from one measurement to another for at least 1 week. After this time, regular analyses of biomass and influent and effluent were carried out for over 42 cycles. The influent, the effluent, and the biomass concentrations in the reactors were analyzed in accordance with APHA (1992). Nitrate, nitrite, and ammonium concentrations were measured as milligram N-NO_3_^−^ per liter, milligram N-NO_2_^−^ per liter, and milligram N-NH_4_^+^ per liter. In addition, these measurements and COD analyses were done during the reactor cycle to observe the changes in the organic and nitrogen compound concentrations over time. All chemical analyses were performed in duplicates. The changes in ammonia concentration followed zero-order kinetic, nitrate concentrations first-order kinetic, while COD changes pseudo-first-order kinetic. The hypothesis on the distribution of each analyzed variable was verified with the Shapiro-Wilk *W* test. The significance of differences between variables was stated with the use of one-way analysis of variance (ANOVA). The homogeneity of variance in groups was checked with the Levene’s test. The significance of differences between analyzed variables was determined with the RIR Tukey test. Differences were found significant at *α* = 0.05.

### Real-time PCR

To assess the activity of bacteria during exposure to SMF, the real-time PCR was done. The AOB activity was assessed based on the *amoA* gene copy number, and overall bacterial community was assesses based on the 16S ribosomal DNA (rDNA) gene copy number. The activated sludge was sampled and fixed according to Cydzik-Kwiatkowska and Wnuk (2011). Samples were taken during the initial 8 h of the cycle, i.e., until the moment, in which the content of pollutants in the cycle stabilized. Samples were stored at −20 °C until RNA was extracted. RNA was isolated with Total RNA kit (A&A Biotechnology), according to the manufacturer’s protocol. The concentration of RNA was measured using NanoDrop Lite (Thermo Scientific). Identical amounts of RNA from each sample was a template to create first-strand complementary DNA (cDNA) using RevertAid™ H Minus First Stand cDNA Synthesis Kit (Fermentas). Real-time amplification of 16S rDNA and *amoA* genes were done in 7500 Real-Time PCR System (Applied Biosystems). Real-time PCR was performed according to Cydzik-Kwiatkowska and Wojnowska-Baryła (2011). The template for the reaction was cDNA. Standard curves for the assessment of 16S rDNA and *amoA* gene copies in the samples were linear within a range of 10^5^–10^10^ for 16S rDNA (*r* ^2^ = 0.9999) and 10^2^–10^7^ for *amoA* (*r* ^2^ = 0.9845).

### Fluorescence In Situ Hybridization

To estimate how SMF affected the abundance of AOB, fluorescence in situ hybridization (FISH) was carried out. Samples of biomass collected from the reactors were fixed, and then microorganisms in the samples were identified as described by Nielsen (2009). The probes NSO190 and EUBmix (EUB338, EUB338-II, EUB338-III) were used; the conditions used for these probes can be found in probeBase (www.microbial-ecology.net/probebase). Vectashield (Vector Laboratories) was used to mount the samples prior to visualization with a Nikon Eclipse epifluorescence microscope (Nikon). The FISH-defined populations (Cy3 or FLUOS labeled) were quantified by image analysis using the ImageJ software (http://rsb.info.nih.gov/ij/), based on examination of at least 30 fields of view for each probe. The standard deviation of all values and probes were 10–15% of the average value indicated.

## Results and Discussion

The study was conducted to assess the influence of SMF on the treatment efficiency and microbial structure and activity of activated sludge. The concentration of biomass was 2950 ± 670 mg MLSS/L in the control reactor and 3420 ± 710 mg MLSS/L in the SMF-exposed reactor. The higher concentration of biomass in the SMF-exposed reactor confirms previous studies concerning the influence of SMF on bacterial communities (Hattori et al. 2001; Wahid et al. 2001). This higher concentration is the result of the SMF that, as a result of absorption and coagulation, promotes creation of bacterial consortia and decreases the concentration of suspended solids in the effluent. Moreover, Jung and Sofer (1997) noted that the magnetic field increased a production of extracellular enzymes which stimulated immobilization of free-living bacteria. The effect of enhanced secretion of extracellular polymeric substances under SMF was used for aerobic granulation (Wang et al. 2012). The authors noted that SMF with a strength of 48 mT shortened the time of aerobic granulation from 41 to 25 days and improved the settling properties of granules. The authors indicated that SMF influenced also the dominant bacteria in granules. Scanning electron microscope observations showed that the bacteria in granules exposed to SMF created dense and compact structures overgrown with coccus bacteria. The structure of granules from the reactor untreated by SMF was loose and dominated by bacillus-like bacteria.

**Fig. 3:**
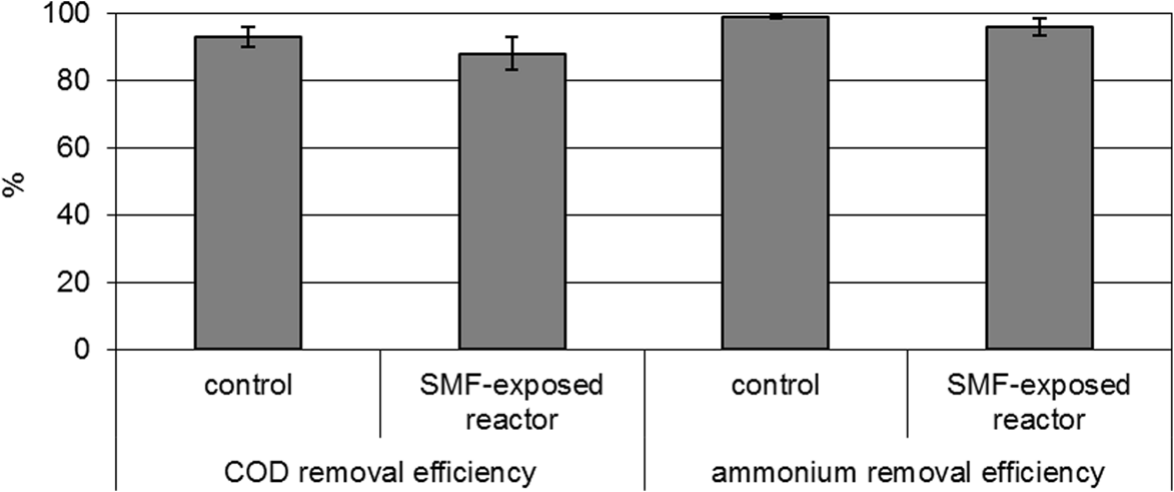
Efficiency of COD and ammonium removal in the control reactor and the SMF-exposed reactor

**Fig. 4:**
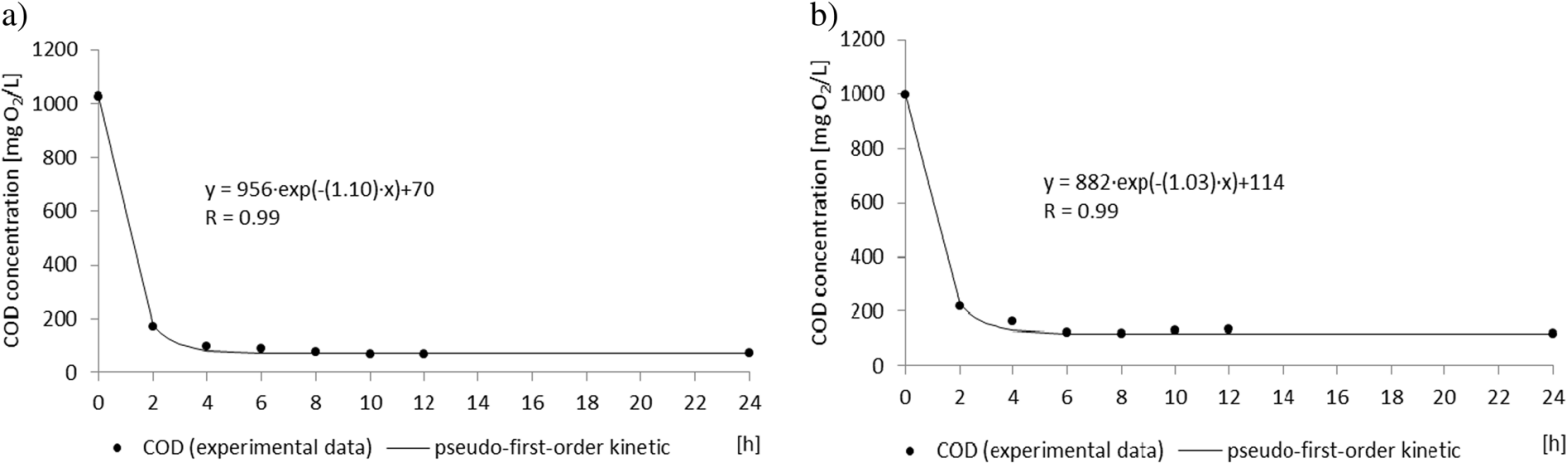
Changes in COD concentrations in the cycle of the control reactor (**a**) and the SMF-exposed reactor (**b**)

The efficiency of COD removal was 93 ± 3% in the control reactor and 88 ± 5% in the SMF-exposed reactor (Fig. 3). In the control reactor, the COD removal rate was 0.36 g/(g MLSS h) and most of COD (830 mg/L) was removed during the first 2 h of the cycle (Fig. 4a). In the SMF-exposed reactor, the COD removal rate was 0.26 g/(g MLSS h) and at the end of the cycle, the COD concentration was 114 mg/L (Fig. 4b). Our results are consistent with results obtained by Rodziewicz et al. (2013). The authors noted that exposure of wastewater and biofilm to the magnetic field did not affect the efficiency of the organic compounds removal. The highest percentage of COD removal, on average 90%, was observed in a rotating biological contactor exposed to magnetic field with induction of 180 mT. Opposite to these results, enhanced COD removal in the presence of magnetic field was noted by Ji et al. (2010). The maximum COD removal ratio was almost 1.5 times higher for mixed bacteria culture exposed to magnetic field (20 mT) than in the control mixed bacteria culture. Moreover, the authors exposed bacteria to magnetic field and then used these bacteria for wastewater treatment and also noted higher COD removal than in not pretreated bacteria. The differences in the presented effects of SMF on COD removal might result from different concentration of COD in the wastewater. Rodziewicz et al. (2013) used the same synthetic wastewater as in the presented paper; however, Ji et al. (2010) used real wastewater with seven times lower COD concentration.

**Fig. 5:**
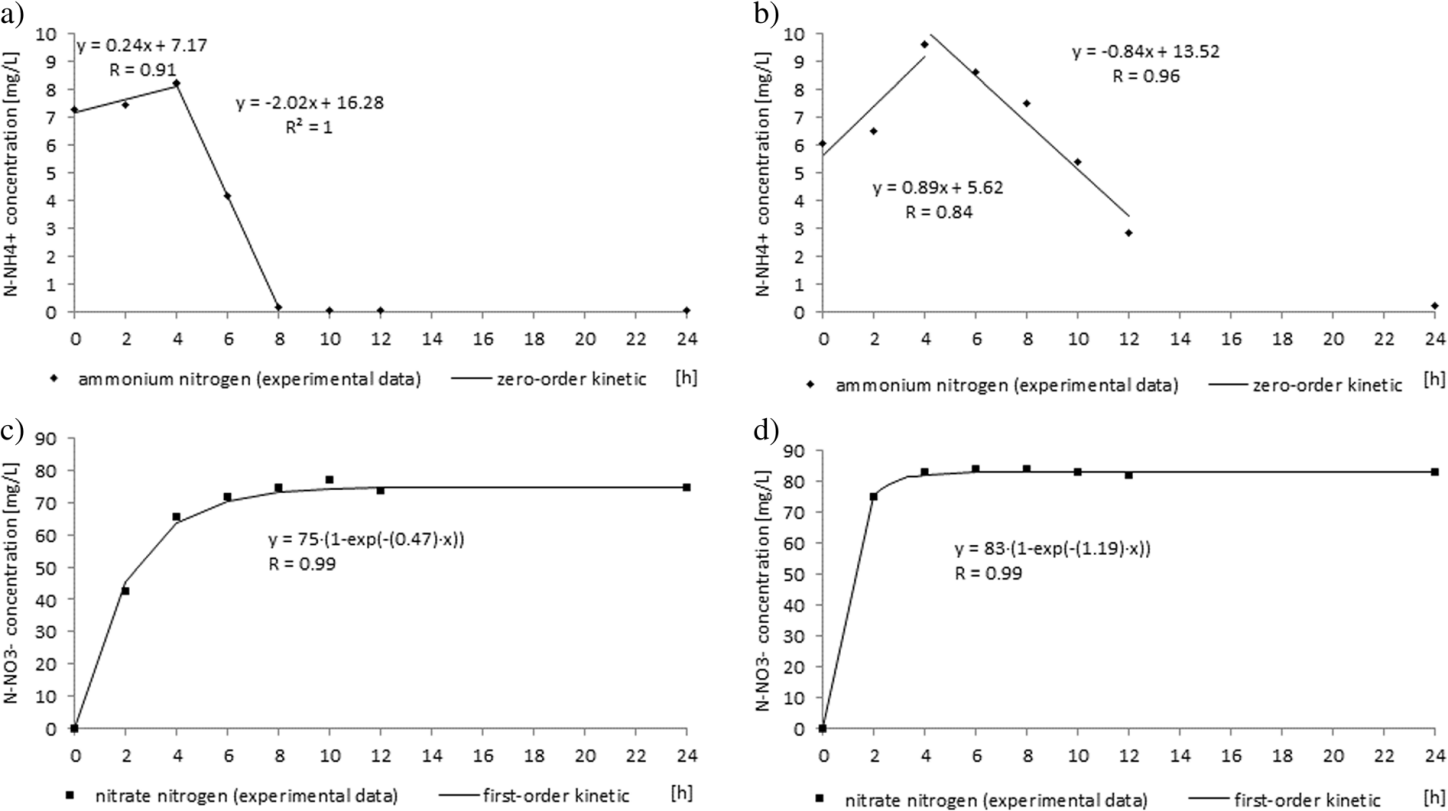
Changes in nitrogen compound concentrations during the cycle. **a** Ammonium concentration in the control reactor. **b** Ammonium concentration in the SMF-exposed reactor. **c** Nitrate concentration in the control reactor. **d** Nitrate concentration in the SMF-exposed reactor

In synthetic wastewater fed into the reactors, organic nitrogen predominated and it was ammonified. In both reactors, ammonium was fully oxidized to nitrates. The efficiency of ammonium removal was high and reached 99 ± 0.5 and 96 ± 2.5% in the control reactor and in the SMF-exposed reactor, respectively (Fig. 3). Rodziewicz et al. (2013) noted increased nitrification under increased magnetic field induction from 60 to 180 mT. Filipič et al. (2015) also observed significantly higher nitrification rates in an SBR exposed to 50 or 30 mT than in the control reactors. Although in the present study the nitrification efficiency was not significantly different in the reactors, nitrification kinetics in the cycle were different. Changes in concentrations of nitrogen compounds during the 24-h reactor cycle are presented in Figure 5. In both reactors, the ammonification and nitrification took place so quickly that ammonium and nitrite concentrations during the cycle were very low. In both reactors, in the first 4 h, the concentration of ammonium increased with the rates of 0.24 mg/(L h) (0.08 mg/(g MLSS h)) in the control reactor and 0.89 mg/(L h) (0.259 mg/(g MLSS h)) in the SMF-exposed reactor (Fig. 4a, b). In the next hours, ammonium was removed and the ammonium oxidation rate was 2.02 mg/(L h) (0.48 mg/(g MLSS h)) in the control reactor and 0.84 mg/(L h) (0.25 mg/(g MLSS h)) in the SMF-exposed reactor. In the control reactor, ammonium was oxidized to nitrates for 8 h. In the reactor exposed to SMF, oxidation of ammonium to nitrates lasted 4 h and after this period, nitrate concentration stabilized at a level of 83 mg/L (Fig. 5c, d). In the control reactor, nitrate production rate was 12 mg/(g MLSS h), while in the SMF-exposed reactor, nitrate production rate was twice higher and reached 29 mg/(g MLSS h). Because the costs of aeration are one of the main positions in the operating costs of wastewater treatment plants, the shortening of time for nitrification by using SMF is economically advantageous. The efficiency of total nitrogen removal was 26 and 20% in the control and the SMF-exposed reactor, respectively.

**Fig. 6:**
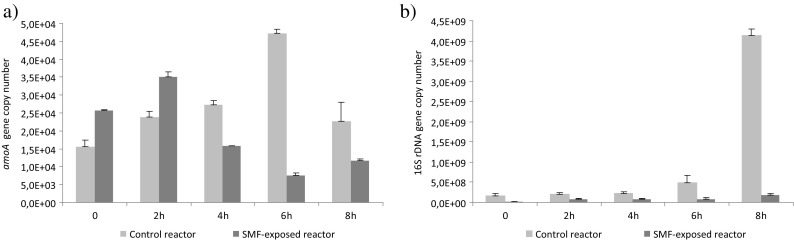
Changes in the *amoA* gene copy number (**a**) and the 16S rDNA gene copy number (**b**) during the reactor cycle

This study investigated the effect of SMF on transcription levels of *amoA* and 16S rDNA genes (Fig. 6). In the control reactor, the copy number of *amoA* gene increased in the first 6 h of the cycle due to the presence of substrate for nitrification. After stabilization of nitrate production, the *amoA* gene copy number in the reactor decreased. In the SMF-exposed reactor, *amoA* gene copy number increased only in first 2 h of the reactor cycle. In this period, the transcription of *amoA* gene was higher in the SMF-exposed reactor than in the control reactor. Higher transcription level of *amoA* gene can be connected with the abundance of AOB that was 7.3% in the control reactor and 12.11% in the SMF-exposed reactor. The higher number of AOB and higher expression of *amoA* gene shortened nitrate production to 4 h. The increased activity of AOB and the higher rates of nitrate production indicate that the SMF also increases the activity of the NOB. In Wang et al.’s (2012) experiment on the influence of SMF on nitrifying granules, FISH analysis showed that the abundances of AOB in the control and experimental reactors were similar but NOB were more abundant in the experimental reactor than in the control reactor. Tomska and Janosz-Rajczyk (2004) and Tomska and Wolny (2008) also found that the nitrification rate of activated sludge exposed to magnetic field was higher than that of the control activated sludge, and the rate of oxygen uptake by NOB was intensified more significantly.

The copy number of 16S rDNA gene in the control reactor slightly increased till the sixth hour of the reactor cycle (Fig. 6b). In the next 2 h, the 16S rDNA gene copy number increased almost 50 times. In the SMF-exposed reactor, the copy number of 16S rDNA gene also increased but in the eighth hour of the reactor cycle, this increase was not as significant. The number of 16S rDNA gene is no longer commonly used to quantify a population’s growth rate in mixed cultures (Blazewicz et al. 2013); therefore, the increase in 16S rDNA resulted from bacterial activity. The increase in bacterial activity during the reactor cycle, in spite of the depletion of substrate, might be a result of bacterial metabolism associated with storage of reserve substances and endogenous process. The activation of bacteria might be as high due to the long reactor cycle, thus long starvation period. Bernhardt et al. (2003) observed that *Bacillus subtilis* produced proteins during famine period that might have protective function against starvation. These proteins have nonspecific but very important function to ensure cell viability in nongrowth state. The lower activity of bacteria in the SMF-exposed reactor could have resulted from the compact structure of the bacterial consortium formed under the influence of a magnetic field. This compact structure limits the oxygen diffusion inside the sludge and lowers the bacterial activity.

## Concluding Remarks

The influence of static magnetic field on the treatment efficiency of wastewater by activated sludge was investigated. In the control and the SMF-exposed reactor, the efficiencies of COD removal of about 90% and nitrification over 95% were noted. In the SMF-exposed reactor, the higher number of *amoA* gene copy and ammonia-oxidizing bacteria and the shortening of time of nitrate production were observed. Because the cost of aeration is the main position in the operating costs of wastewater treatment plants, the shortening of time for nitrification by using SMF is economically advantageous.
